# Impact of temporal fluctuations in optical defocus on visual acuity: Empirical results and modeling outcomes

**DOI:** 10.1167/jov.23.3.14

**Published:** 2023-03-27

**Authors:** Sabyasachi Goswami, Shrikant R. Bharadwaj

**Affiliations:** 1 Brien Holden Institute of Optometry and Vision Sciences, L V Prasad Eye Institute, Hyderabad, Telangana, India; 2 Prof Brien Holden Eye Research Centre, Hyderabad Eye Research Foundation, L V Prasad Eye Institute, Hyderabad, Telangana, India; 3 Brien Holden Institute of Optometry and Vision Sciences, L V Prasad Eye Institute, Hyderabad, Telangana, India; 4 Prof Brien Holden Eye Research Centre, Hyderabad Eye Research Foundation, L V Prasad Eye Institute, Hyderabad, Telangana, India

**Keywords:** defocus, focus tunable lens, Fourier optics, template matching, temporal averaging

## Abstract

Optical defocus in human eyes is seldom steady during naturalistic steady-state viewing. It fluctuates by 0.3 to 0.5 diopters (D) from accommodative microfluctuations and by 1.5 to 2.5 D in dysfunctions such as spasm of near reflex, both with ≤2 Hz low-pass frequency spectra. This study observed losses in monocular visual acuity of cyclopleged adults who encountered varying amplitude (0.25–2.0 D) and temporal frequency (0.25–2.0 Hz) combinations of sinusoidal defoci induced using an electrically tunable lens. Visual acuity, recorded for 300-ms flashes of Sloan optotype presentation using the method of constant stimuli, deteriorated with defocus amplitude at a rate steeper for lower than higher temporal frequencies. A template matching model of acuity, incorporating optical and neural low-pass filters, neural noise, and a cross-correlated decision operator, showed the best match with empirical data when acuity was governed by the minimum defocus available during optotype display. This criterion minimized acuity loss for higher temporal frequencies due to the increased probability of zero-defocus encounters within the presentation duration. Other decision criteria such as defocus averaging across the entire or parts of the presentation duration yielded less satisfactory results. These results imply that vision loss in humans encountering broadband time-varying defocus is dictated by the dominant low frequencies, with higher frequencies largely compensated using the least defocus decision strategy.

## Introduction

Optical defocus is an inherent feature of our naturalistic visual experience. Objects that fall outside the eye's current plane of focus result in myopic or hyperopic defoci on the retina (Atchison & Charman, 2010; Charman, 2008; Charman, 2011; Wilson, 2017). This defocus, either in isolation or in combination with other higher-order aberrations, produces a significant deterioration of retinal image quality (Applegate, Sarver, & Khemsara, 2002), leading to loss of visual functions (Lombardo & Lombardo, 2010), functional vision (Colenbrander, 2010), and quality of life (Colenbrander, 2010). A common assumption made in previous studies of the nature mentioned above is that the retinal image defocus and, consequently, the vision loss are stable over time for a given accommodative and fixation state (Charman, 2008; Charman, 2011). This study is concerned with instances where this assumption may be violated and where the impact of time-varying defocus on vision cannot be ignored.

Two instances wherein the visual system encounters a time-varying defocus signal are relevant in this context. First, during steady-state viewing, the refractive power of the eye varies continuously with a frequency bandwidth of <2 Hz and amplitude of ∼0.3 diopters (D) due to the physiological microfluctuations of accommodation (Charman & Heron, 1988; Charman & Heron, 2015). Second, refractive power fluctuations that are similar in bandwidth to the accommodative microfluctuations but with much larger amplitudes (∼1.5–2 D) are also observed in a binocular vision dysfunction referred to as the spasm of near reflex (Bharadwaj, Ravisankar, Roy, & Satgunam, 2021; Bharadwaj, Roy, & Satgunam, 2020). Surprisingly, very little is known about the impact of these temporal fluctuations in optical defocus on visual functions in humans, even though their negative impact on the image quality of general optical systems was recognized decades ago (Lohman & Paris, 1965). Defocus fluctuations arising from accommodative microfluctuations are thought to be within the perceptual depth of focus and may not result in an experience of blur (but see Winn, Charman, Pugh, Heron, & Eadie, 1989; Charman & Heron, 1988; Charman & Heron, 2015). On the other hand, defocus fluctuations in the spasm of near reflex do produce a significant loss of visual acuity and elicit subjective impressions of vacillating image quality in patients (Bharadwaj et al., 2020; Roy, Bharadwaj, Patil-Chhablani, & Satgunam, 2021).

Few recent studies have induced temporal variations in defocus to study their impact on high contrast visual acuity (Ampolu, Yarravarapu, Satgunam, Varadharajan, & Bharadwaj, 2022; Bartuzel, Iskander, Marin-Franch, & Lopez-Gil, 2019; Dorronsoro et al., 2019). Bartuzel et al. (2019) reported an improvement in visual acuity when induced static myopic defocus was combined with 50-Hz trapezoidal variations in temporal defocus relative to when the temporal fluctuations were absent. Dorronsoro et al. (2019) applied such fluctuations for temporal multiplexing of defocus as a presbyopia management strategy. The patterns of temporal defocus used in both studies were far removed from what is experienced with physiological accommodative microfluctuations (Charman & Heron, 2015) or with the spasm of near reflex (Bharadwaj et al., 2020). More recently, Ampolu et al. (2022) reported loss of visual acuity in healthy controls induced by the pattern of temporal defocus experienced in the spasm of near reflex. The visual acuity loss was greater for static myopic defocus than for temporal variations in defocus about emmetropic refraction, but the loss compounded when static and temporal variations in defocus were present in combination. Although this study offered insights into the visual acuity loss with the spasm of near reflex, no attempt was made to disentangle the impact of frequency and amplitude of defocus on visual acuity (Ampolu et al., 2022). Given these shortcomings, the impact of temporal variations of defocus on the visual acuity of human observers remains incompletely understood in the literature.

This issue was systematically addressed here in two ways. First, empirical evidence for the impact of temporal variations in defocus on visual acuity loss was determined by inducing varying combinations of sinusoidal oscillations in defocus in otherwise visually normal adults over the amplitude and frequency range typically observed with accommodative microfluctuations (Charman & Heron, 2015) and spasm of near reflex (Bharadwaj et al., 2020). Second, a computational template matching model of acuity was constructed to provide the underlying theoretical framework for the empirical data. This analysis offered insights into the strategies that may be adopted by the visual system to optimize performance in the presence of such temporal variations in retinal image defocus.

## Methods

### Subjects

Thirteen subjects (20 to 26 years of age; five males and eight females) with uncorrected visual acuity of 20/20 or better, with manifest non-cycloplegic spherical equivalent refractive error ≤ 0.50 D, and free of any ophthalmic pathology, as deemed by a comprehensive eye examination, were recruited for this study from among the staff and student pool of the L V Prasad Eye Institute (LVPEI), Hyderabad. The mean (±1 *SD*) post-cycloplegic spherical equivalent refractive error was +0.31 ± 0.15 D across all of these subjects. The study protocol adhered to the tenets of the Declaration of Helsinki, and it was approved by the Institutional Review Board of LVPEI. The study protocol was initiated after each subject signed a written informed consent form. Subjects allergic to cycloplegic eye drops and those unavailable for repeated testing were excluded. Of the 13 subjects recruited, only one was trained as a psychophysical observer; the others were naïve to such procedures. All subjects were naïve to the purpose of the present experiment.

### Visual acuity assessment

Monocular logMAR visual acuity was measured in a semi-dark room using the method of constant stimuli psychophysical procedure written in MATLAB (MathWorks, Natick, MA). One eye of the subject was randomly chosen for testing, and the fellow eye was occluded. Subjects identified single Sloan optotypes presented on a cathode-ray tube monitor (1024 × 768-pixel resolution) for 300 ms each from a 2-meter viewing distance using the Psychtoolbox interface for MATLAB (Brainard, 1997). The psychometric function was generated by presenting 11 different optotype sizes, 10 times each in random order, resulting in a total of 110 presentations for each test condition. For each presentation, one of the 10 Sloan optotypes of a given size was randomly chosen from the image database. The subjects were not aware that only 10 out of 26 English alphabets were to be presented, thus rendering the task a 26-AFC procedure, with a chance level of 3.85%. The resultant data were fitted with a cumulative Gaussian distribution function, with the mean and standard deviation of the function kept as free parameters. The fit was optimized using the fmin search function in MATLAB based on the maximum likelihood–based Nelder–Mead simplex method. The 51.9% correct response of the psychometric function was considered as the threshold visual acuity, and the slope of psychometric function was taken as a measure of task precision (steeper slopes = greater task precision). Given that the visual acuity was measured over a range of defoci, the optotype sizes were chosen over the range of −0.5 logMAR (20/6.3) to 1.3 logMAR (20/399) in 0.1-logMAR steps such that they represented the entire range of psychophysical performance. The center value of the optotype size range was selected from the clinically expected acuity value for a given magnitude of static defocus. Five optotype sizes above and below this center value were chosen as the test range to construct the psychometric function. If the first 25% of the trials in the experimental session resulted in all correct or all incorrect responses from the subject, the session was aborted and reinitiated by decreasing or increasing the center value of the optotype size, respectively, by 0.2 logMAR units.

Visual acuity was measured on each subject without any induced defocus (baseline) and with sinusoidal temporal variations in defocus, with amplitudes of 0.25 D, 0.5 D, 1.0 D, and 2.0 D, each at temporal frequencies of 0.25 Hz, 0.5 Hz, 1.0 Hz, and 2.0 Hz. A total of 17 visual acuity measurements were obtained on each subject: baseline + (4 amplitudes × 4 frequencies) = 17 measurements. Also, the order of testing of each amplitude and frequency combination of defocus was randomized across subjects. All measurements commenced after the subject's eye was cyclopleged, 40 minutes after instillation of 1% cyclopentolate hydrochloride eye drops, using standard clinical protocols. Cycloplegia ensured that the eye's innate accommodative microfluctuations, pupil size, and the associated changes in lower- and higher-order wavefront aberrations of the eye remained fixed throughout the experiment and did not interfere with the experimentally induced temporal defocus fluctuations (Hofer, Artal, Singer, Aragon, & Williams, 2001; Johnson, Post, & Tsuetaki, 1984). Both of these control measures ensured that the visual acuity measurements reported here were free of any ambiguity arising from the innate dynamic alternations in the eye's retinal image quality. Elimination of accommodative microfluctuations and persistence of pupil dilation after cycloplegia was verified for every subject by monitoring the refraction profile of both eyes periodically (at an interval of ∼90 minutes) during the experiment using a dynamic, eccentric, infrared photorefractor (PowerRef 3; Plusoptix GmbH, Nuremberg, Germany) (Bharadwaj et al., 2020). If required, additional cycloplegic eye drops were instilled to ensure persistent cycloplegia. Any residual refractive error after cycloplegia remained uncorrected during the experiment to avoid adding additional lenses in the optical path of the subject during empirical testing of visual acuity.

### Temporal defocus stimulation

Temporal fluctuations in optical defocus were induced using a commercially available electrically focus tunable lens (ETL, EL-16-40-TC; Optotune AG, Dietikon, Switzerland), procured through a third-party vendor (Edmund Optics, Bengaluru, Karnataka) (see Appendix A for static and dynamic calibration of ETL). The ETL was placed at a vertex distance of ∼20 mm before the subject's right eye. Appropriate correction factor was built into the current input in order to account for the effective power produced by the ETL at this vertex distance. A control experiment was performed on six subjects to ensure that visual acuity measurements for a given magnitude of optical defocus were similar for those induced with conventional trial lenses and those with the ETL at comparable vertex distances (see Appendix A for details). There was no synchronization between the defocus fluctuations produced by the ETL and the optotype presentation during psychophysical testing. Therefore, the magnitude and sign of defocus experienced for a given optotype and size were completely unpredictable during visual acuity testing. This was done so to mimic real-world human experience, wherein the refractive fluctuations from accommodative microfluctuations or spasm of near reflex are independent of what is observed in the outside world. Each experimental run took ∼8 minutes to complete, and the total experiment took ∼4 hours (including break times and cycloplegia effect times) to complete in each subject. These measurements were obtained over several sessions, with adequate breaks to avoid subject boredom and fatigue.

### Template matching model of acuity

**Figure 1.**
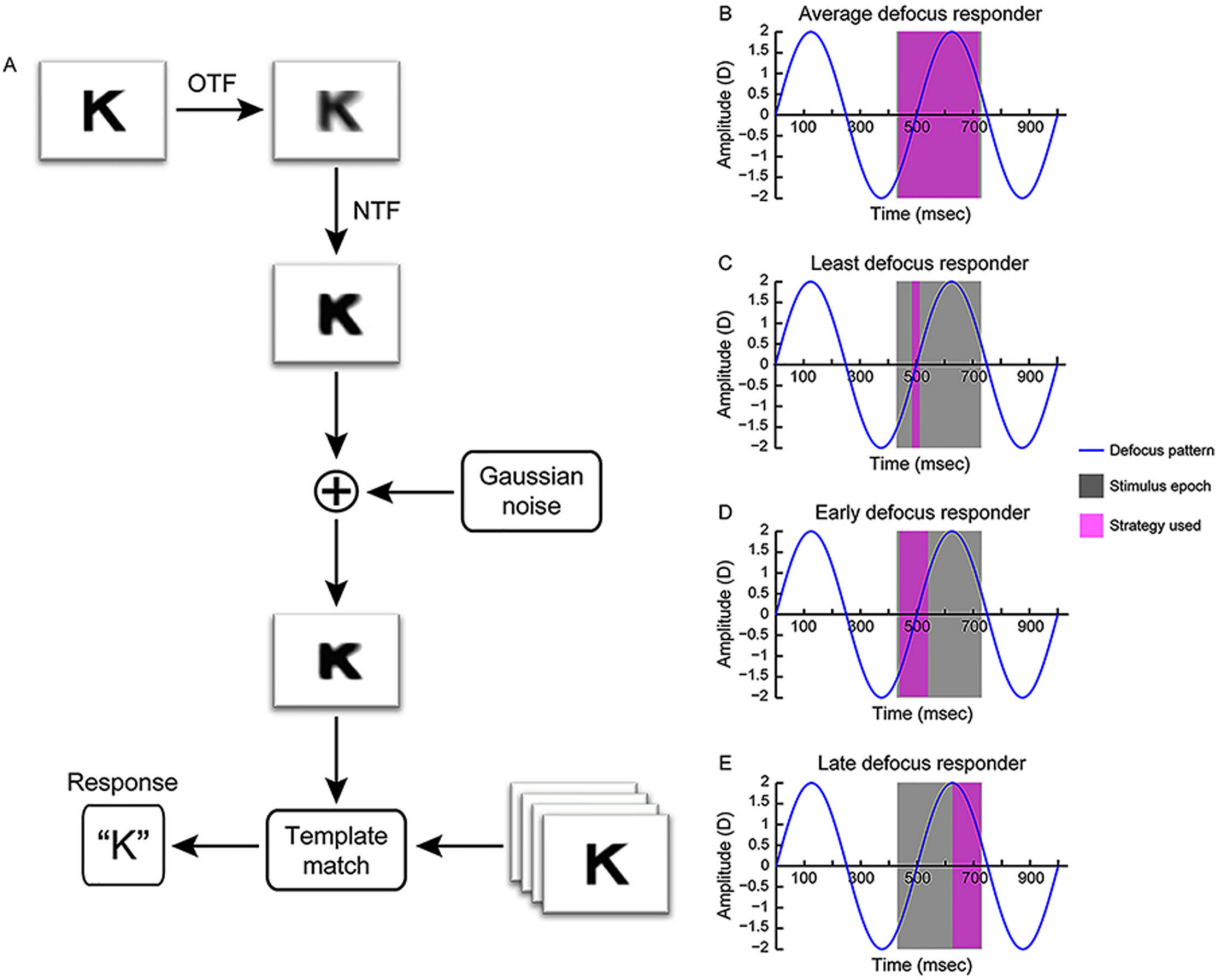
(A) Schematic of the template matching model for acuity used to determine the impact of temporal defocus variations on visual acuity. (B–E) Schematics of the four different decision strategies tested in this study to optimize performance in the presence of temporally varying defocus. The blue curve shows a sinusoidally varying defocus signal with an amplitude of ±2 D and 2-Hz frequency. The gray band shows a 300-ms-long stimulus epoch, appearing from 430 ms to 730 ms in this instance. During the test, the stimulus epoch randomly occurred at any location in the sinusoidal oscillation. The pink band shows the four different temporal averaging strategies that were tested in this study. A fifth strategy involving random combinations of the four strategies was also tested but is not shown in this figure.

A template matching model of acuity was constructed to provide the underlying theoretical framework for the empirical results described above and to explore putative strategies used by the visual system to optimize performance in the presence of temporal variations in optical defocus (Figure 1A). The model was inspired by the one developed by Watson and Ahumada (2008) for predicting the visual acuity of humans from the wavefront aberrations of their eye. The model essentially contained all the elements of the Watson and Ahumada model but with two key functional differences (Figure 1A). First, unlike Watson and Ahumada (2008), the goal of the present model was not to match the output with the visual acuity of individual subjects but to describe the overall trends in the data with different amplitude and frequency combinations of temporal defoci. Specific model parameters (e.g., neural noise) were therefore not optimized in the present model to fit the empirical data, unlike Watson and Ahumada (2008). Second, the Watson and Ahumada model predicted visual acuity for static optical defocus but the present model had temporal variations in optical defocus as its input. The output of the present model was, however, tested and validated for a range of static optical defoci before it was applied for temporal variations in defocus (see Appendix B for details). The key steps in building the present model are described below.

1. *Creation of optotypes*—The optotypes used for the template matching analysis were from the same database of images used for empirical testing. Each grayscale image was 255 × 255 pixels in size with the resolution of 92 dpi. Like empirical testing, the database contained images of all 10 Sloan optotypes ranging from −0.5 logMAR units to +1.3 logMAR units in 0.1-logMAR unit steps (Figure 1A).
2. *Filtering optotypes with the optical transfer function* (*OTF)*—Spatial resolution is primarily limited by the low-pass filtering properties of the eye's optics (Chen, Makous, & Williams, 1993; Van Meeteren, 1974). In this analysis, template images were filtered with the two-dimensional OTF derived from the population-average higher-order aberrations of the eye for 5-mm pupil diameter (Cheng et al., 2004), using standard Fourier optics techniques (Thibos, Hong, Bradley, & Applegate, 2004) (Figure 1A). This was done to account for the image quality loss that may be inherent to the habitual viewing experience of humans from very early on in development, and they may have a strong influence on the formation of the neural template (Candy, Wang, & Ravikumar, 2009; Wang & Candy, 2005). Test images were obtained by filtering the optotypes using the same technique but with varying magnitudes of the induced defoci ($Z_{2}^{0})$, as required by the experiment, for 5-mm pupil diameter (Thibos, Hong, Bradley, & Cheng, 2002).
3. *Filtering optotypes with the neural transfer function* (*NTF)*—Images available for visual processing were additionally low-pass filtered by the NTF (Watson & Ahumada, 2008). In the present analysis, this was achieved by filtering both the template and test images using two-dimensional NTFs described recently by Hastings, Marsack, Thibos, and Applegate (2020) for a 25-year-old subject and for a retinal illuminance level of 1100 trolands (Figure 1A). To these images, an additional zero-mean white Gaussian noise with variance of 0.144 units was added to represent neural noise (Figure 1A) (Watson & Ahumada, 2008). This variance level produced the best match of the Watson and Ahumada model with empirical data.
4. *Template matching*—The template and test images were matched using the normxcorr2.m cross-correlation–based template matching operation in MATLAB (Figure 1A). For every test image presented, the matching was performed across all 10 template images of the corresponding logMAR size, and the ones with normalized cross-correlation values ≥ 0.95 were considered to be potential matches (Figure 1A). If more than one template image met the cross-correlation criterion, the match with the highest value of cross-correlation was considered to be the response (Figure 1A). If the highest value of cross-correlation across matches differed only at the third decimal level, one of the matches was picked randomly as the response (Figure 1A). Care was taken to ensure that the matches happened only in the region containing the optotype feature, especially for small optotype sizes that contained large white regions devoid of any information.
5. *Psychophysical procedure*—The template matching model was automated to generate a psychometric function based on the method of constant stimuli, such as empirical data collection. For every presentation of the test image, if the optotype identified in step 4 equaled the one presented, the match was considered to be the correct response. This procedure was followed for all 110 test target presentations to generate the psychometric function. All other procedures pertaining to deriving acuity and task precision from the psychometric function were identical to empirical data collection. Variance in the percentage of correct response obtained for each optotype size in the psychometric function was derived using standard formula described in Macmillan and Creelman (1991). The overall variance of acuity and task precision for each defocus amplitude and frequency combination was obtained by performing a Monte Carlo simulation of the psychometric function 100 times using these variance values. The mean acuity and the slope of the psychometric functions obtained across these repetitions were then compared against empirical data.

### Temporal defocus signal

The template matching model for static defocus is relatively straightforward to implement, for the defocus signal remains time invariant and the database containing the defocused images is constant with a given presentation epoch (300 ms, in this case). Implementing this model for temporally varying defocus is, however, more involved because the magnitude of optical defocus varies with the amplitude and frequency of the sinusoidal signal, and its impact on visual acuity will depend on a combination of (a) the optotype size that is presented, (b) the phase of the defocus cycle in which the optotype was presented, and (c) the strategy used by the visual system to optimize recognition of this optotype within the presentation epoch (Figures 1B to 1E). Further, the defocus experienced by the retina will have different dynamics depending on the amplitude and frequency of the sinusoidal signal. The rate of change of defocus will be highest for the combination with ±2 D amplitude at 2 Hz and least for the combination with ±0.25 D amplitude at 0.25 Hz. Thus, the chances of experiencing a zero-defocus crossing within the 300-ms presentation epoch will increase with the frequency of temporal fluctuations across all amplitudes (see Appendix C for details). Its impact on acuity measurements is thus likely to vary with the frequency of the temporal defocus fluctuations and the strategy used to optimize acuity with such fluctuations. Finally, like static defocus, the impact of all of these variables will be greater for smaller optotypes than for larger optotypes.

**Figure 2.**
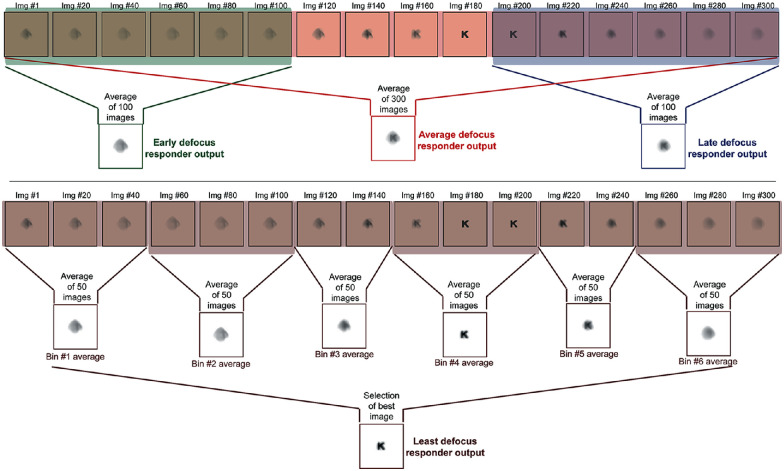
Schematic of the averaging process adopted for the average defocus, least defocus, early defocus, and late defocus strategies in the template matching model of visual acuity. The upper panel shows the schematic for the average, early, and late defocus strategies, and the lower panel shows the schematic for the least defocus strategy. In all strategies, retinal images were generated for defocus values in 1-ms intervals, resulting in 300 defoci values within the presentation epoch. All 300 images were averaged in the average defocus strategy, and the first 100 and last 100 images were averaged in the early and late defocus strategies, respectively, for the template matching process. In the least defocus strategy, the 300-ms epoch was divided into six 50-ms bins, and the images within each bin were averaged and then selected based on the least defocus for the template matching process.

Given these complexities, the template matching model adopted the following paradigm to generate psychometric functions with different amplitude and frequency combinations of defocus tested in this study. For every iteration of the psychophysical procedure, the optotype and its size and the 300-ms presentation epoch of the test image were randomly assigned to a location in the sine-wave cycle. The defocus values within this presentation epoch were sampled in 1-ms intervals, and 300 defocused images of the optotype, each corresponding to the millisecond of the sample, were generated as described in step 2 above. This process was repeated for all 110 iterations of the psychophysical procedure described in step 5, resulting in a total of 33,000 defocused images of varying optotypes and their sizes for a given combination of defocus amplitude and frequency. The same procedure was followed for all combinations of defocus amplitude and frequency tested in this study. The following five decision-making strategies were then tested: the average defocus responder strategy (Figure 1B), the least defocus responder strategy (Figure 1C), the early defocus responder strategy (Figure 1D), the late defocus responder strategy (Figure 1E), and a potpourri of the four strategies decided on a trial-by-trial basis (the mixed responder strategy). In the average defocus responder strategy, all 300 defocused images within the presentation epoch were averaged and chosen for template matching (Figures 1B and 2). In the least defocus responder strategy, the 300-ms epoch was divided into six 50-ms bins, and the defocused images within each bin were averaged (Figures 1C and 2). The least defocus value among the six bins was identified, and the image corresponding to this bin was then chosen for template matching (Figures 1C and 2). In the early (Figures 1D and 2) and late (Figures 1E and 2) defocus responder strategies, the defocused images corresponding to the first 100 ms and last 100 ms, respectively, were averaged. In the mixed responder strategy, the algorithm randomly chose one of the four strategies on each trial of the psychophysical procedure with equal probability.

## Results

Data were successfully collected from all study participants. Photorefraction measurements obtained periodically during the experiment showed no steady-state fluctuations of accommodation, indicating persistence of cycloplegia throughout the experiment. The Kolmogorov–Smirnov test indicated that the visual acuity obtained from the empirical study followed a normal distribution; therefore, a similar assumption was made for all of the simulated data. Parametric two-factor, repeated-measures analyses of variance (ANOVAs; defocus amplitude × frequency) were used for statistical analysis. Pairwise comparison was performed using Bonferroni post hoc tests, with appropriate *p*-value correction for multiple comparisons.

### Visual acuity loss with temporal defocus

**Figure 3.**
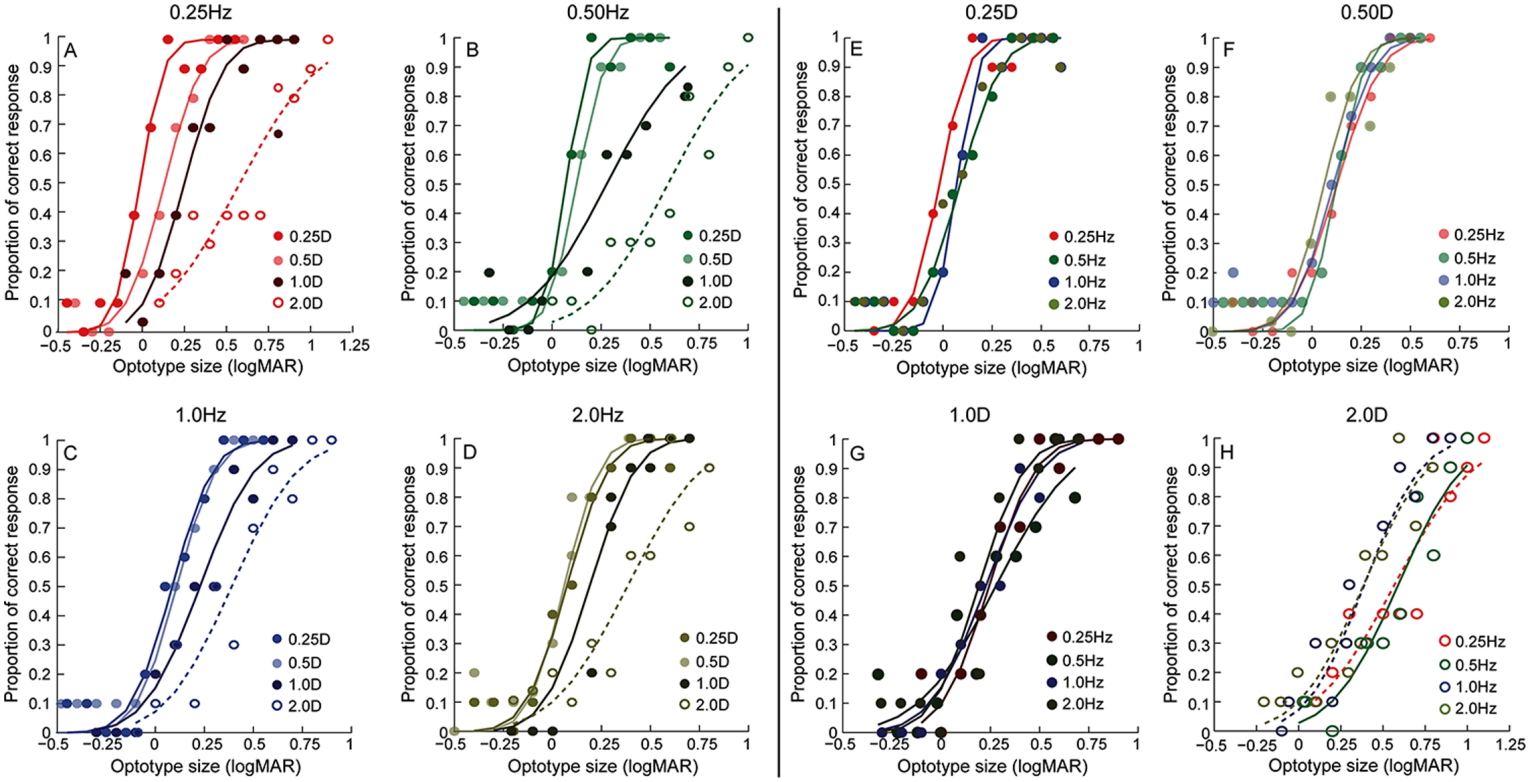
Psychometric functions of the proportion of correct response plotted as a function of optotype size for all 16 amplitude and frequency combinations of defocus for a representative subject that participated in this study. (A–D) Data for the 0.25-Hz, 0.5-Hz, 1.0-Hz, and 2.0-Hz frequencies of defocus fluctuation, respectively. In each panel, four psychometric functions corresponding to the four defocus amplitudes tested in this study are plotted. The abscissa scale for the 0.25-Hz frequency of defocus fluctuation is wider than that of the other three frequencies, reflecting the greater loss of acuity in this condition than in others. (E–H) The same data regrouped for the four temporal frequencies for each defocus amplitude. The abscissa scale for the 2.0 D of defocus amplitude is wider than that of the other three amplitudes, reflecting the greater loss of acuity in this condition than in the others.

Figure 3 shows the psychometric functions of the proportion of correct responses plotted against the size of the optotype in logMAR units from one representative subject for all 16 amplitude and frequency combinations of temporal defocus. The data in this figure are plotted in two ways to clearly demonstrate three trends in the data (Figures 3A to 3D); the data obtained across the four defocus values are grouped for a given temporal frequency, and, in Figures 3E to 3H, the data are regrouped across the four temporal frequencies for a given defocus value. First, for any given temporal frequency, the psychometric functions progressively shifted to the right along the abscissa with increasing defocus amplitude, indicating worsening of visual acuity (Figures 3A to 3D). Second, the horizontal spreads in the psychometric functions were greater for lower temporal frequencies than for higher temporal frequencies, indicating that the range of acuity loss with increasing defocus was inversely proportional to the temporal frequency of fluctuation (Figures 3A to 3D). Third, the slope of the psychometric function also became shallower with increasing defocus amplitudes, indicating worsening of task precision (Figures 3E to 3H).

**Figure 4.**
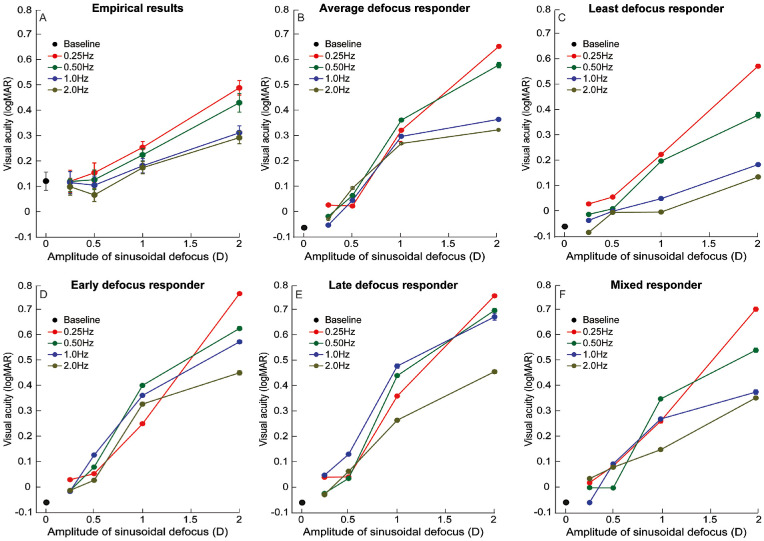
Mean (±1 *SEM*) logMAR visual acuity, estimated from the 51.9% of correct responses in the psychometric function and plotted as a function of the amplitudes of sinusoidal defocus for all four temporal frequencies tested in this study obtained from humans (A) and from the template matching analysis for the five strategies described in the text: average defocus responder (B), least defocus responder (C), early defocus responder (D), late defocus responder (E), and mixed responder (F). Visual acuity obtained at baseline without any induced defocus is also included in this plot.

The representative trends in Figure 3 were confirmed in the mean (±1 *SEM*) visual acuity data obtained for all amplitude and frequency combinations of defocus tested (Figure 4A). The mean post-cycloplegia baseline visual acuity across all participants was 0.12 ± 0.04 logMAR units (Figure 4A). Two-factor, repeated-measures ANOVA showed statistically significant main effects of both temporal frequency and defocus amplitude on visual acuity (*p* ≤ 0.01, for both) (Figure 4A). The interaction between frequency and amplitude was also statistically significant (*p* < 0.001), indicating that the loss of visual acuity with different amplitudes of defocus was non-uniform across temporal frequencies (Figure 4A). Post hoc analysis showed that the visual acuity with 2 D of induced defocus was statistically significantly different from the no-defocus condition across all frequencies (*p* < 0.001), with the loss being relatively largest for the 0.25-Hz frequency and least for the 2-Hz frequency (Figure 4A). The visual acuity loss with 1 D of defocus was statistically significantly different from the no-defocus condition only for 0.25-Hz and 0.5-Hz temporal frequencies (*p* < 0.001, for both) but not for the 1-Hz and 2-Hz temporal frequencies (*p* ≥ 0.12, for both) (Figure 4A). The visual acuity losses with the 0.25-D and 0.5-D defoci were not significantly different from the no-defocus condition across all frequencies (*p* ≥ 0.1) (Figure 4A). Interestingly, the visual acuity with 0.5-D defocus at 2-Hz temporal frequency was significantly better than the no-defocus condition by a mean value of 0.51 ± 0.07 logMAR units (*p* = 0.02) (Figure 4A). Such an improvement in visual acuity at this defocus amplitude was not observed for any other temporal frequency tested in this study (Figure 4A).

Figures 4B to 4F show the mean (±1 *SEM*) visual acuity obtained for the five strategies tested using the template matching model described in Figure 1. The mean (±1 *SEM*) simulated visual acuity obtained from the template matching model was −0.03 ± 0.01 logMAR units for the no-defocus condition (Figures 4B to 4F). The two-factor, repeated-measures ANOVA analysis indicated a main effect of defocus amplitudes on visual acuity across all five strategies (*p* < 0.001, for all) (Figures 4B to 4F). The main effects of temporal frequency and interaction between amplitude and frequency on visual acuity were statistically significant only for the average defocus responder, least defocus responder, and the mixed defocus responder strategies (*p* < 0.001, for all) (Figures 4B, 4C, and 4F). Post hoc analysis for pairwise comparison of defocus amplitudes indicated statistically significant loss of acuity only for the 1.0-D and 2.0-D stimuli (*p* < 0.001, for all) but not for the 0.25-D and 0.5-D stimuli across all of these strategies (*p* ≥ 0.3, for all). Post hoc analysis for pairwise comparison of temporal frequencies showed statistically significant differences across all four frequencies in the average defocus responder and mixed defocus responder strategies (*p* < 0.01, for both) (Figures 4B and 4F). For the least defocus responder strategy, significant differences in acuity were observed only between the lower two frequencies (0.25 Hz and 0.5 Hz) and higher two frequencies (1.0 Hz and 2.0 Hz) (*p* < 0.01) but not within the lower and higher frequencies (*p* > 0.1) (Figure 4C). The early and late defocus responder strategies did not show a significant main effect of temporal frequency (*p* ≥ 0.3) or interaction between amplitude and frequency (*p* ≥ 0.7) (Figures 4D and 4E).

### Precision of performance with temporal defocus

**Figure 5.**
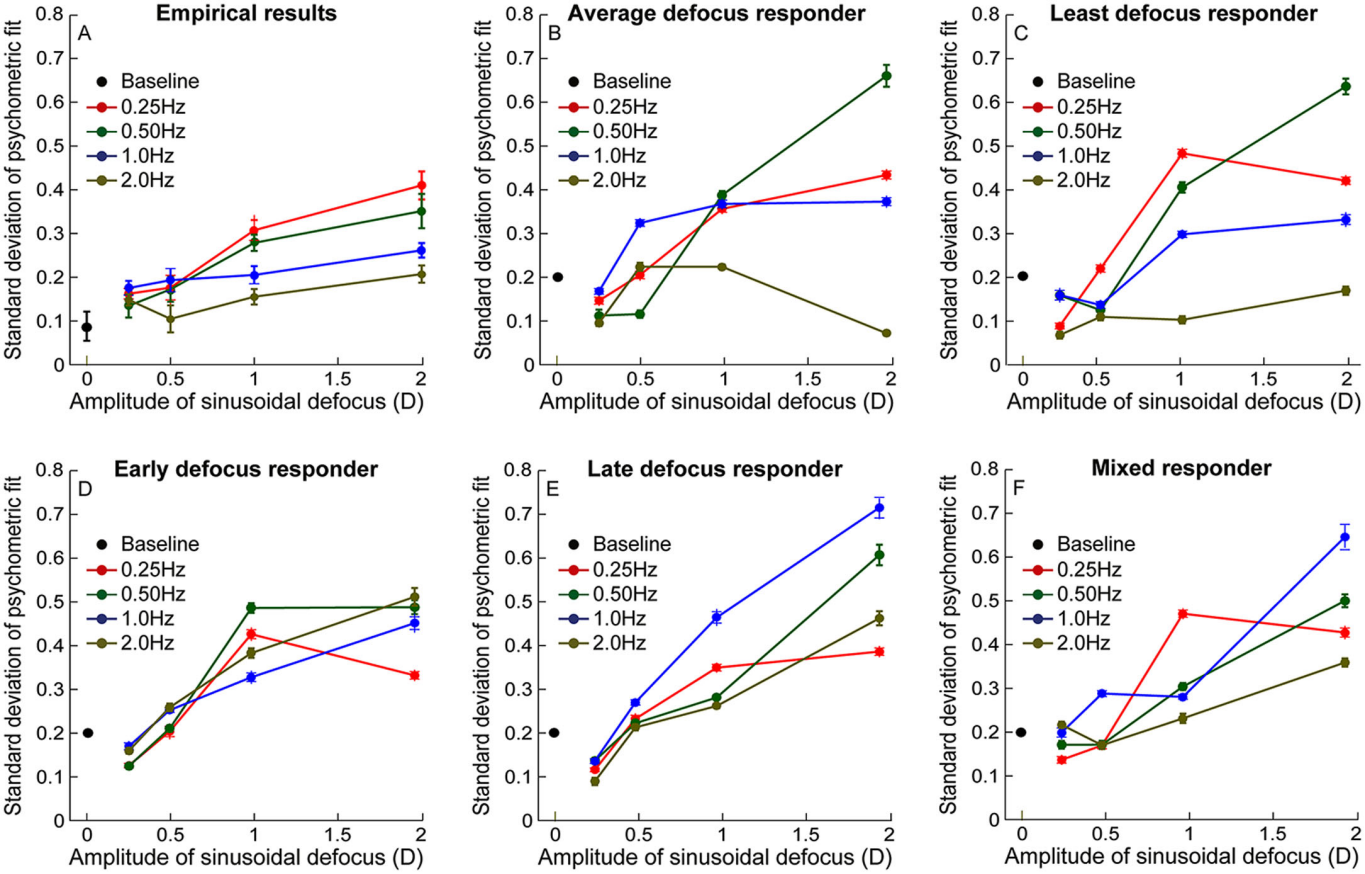
Mean (±1 *SEM*) slope of the psychometric function (i.e., standard deviation parameter of the cumulative Gaussian psychometric function fit) plotted as a function of the amplitude of sinusoidal defocus for all four temporal frequencies tested in this study. (A) Data obtained from humans. (B–F) The template matching analysis for the five strategies described in the text. All other details are the same as in Figure 4.

Figure 5 plots the mean (±1 *SEM*) slope of the psychometric function for the different frequency and amplitude combinations of sinusoidal defoci obtained from the empirical data (Figure 5A) and model simulations (Figures 5B to 5F). In general, the slope of the psychometric function flattened with increasing defoci amplitudes across all four temporal frequencies (Figure 5A). This gradation in flattening was most evident for the 0.25-Hz and 0.5-Hz temporal frequencies, but not so much for the 1-Hz and 2-Hz frequencies, all relative to baseline (Figure 5A). Two-factor, repeated-measures ANOVAs for the empirical data showed significant main effects of both temporal frequency and defocus amplitude and significant interaction between the two factors on the slope of the psychometric function (*p* ≤ 0.001, for all) (Figure 5A). Post hoc tests revealed that the slope values of the 1-D and 2-D defocus conditions were significantly different from baseline across all frequencies (*p* ≤ 0.004, for both), and those of 0.25-D and 0.5-D were not significantly different from the baseline condition (*p* ≤ 0.31, for both). Like acuity, the slope of the psychometric function marginally steepened only for 0.5-D defocus at 2-Hz temporal frequency, relative to the no-defocus conditions (Figure 5A), but the difference in slope values were not statistically significant (*p* = 0.72).

### Comparison of empirical results with model simulations

The most prominent trend in the empirical data (i.e., the rate of acuity loss with defocus was inversely proportional to the temporal frequency of fluctuation) was compared with the five model simulations to determine the most probable strategy used by the visual system to generate the empirical results (Table 1). The data for logMAR acuity as a function of defocus amplitude were fit with a linear regression equation for each temporal frequency, and the slope values of this regression equation were compared among the empirical data and model strategies (Table 1). Linear regression fits resulted in coefficients of determination (*R*^2^) ≥ 0.8 for all temporal frequencies in the empirical data and model simulations (Table 1). The slope values of the linear regression equations were closest to the empirical data in the least defocus responder strategy, followed by the average defocus strategy, mixed responder strategy, and then by the early and late defocus strategies (Table 1). The *y*-intercepts of the empirical data were poorer than 20/20 acuity in the empirical data, but they were better than 20/20 in all of the model simulations (Table 1). Unlike the slope values, there were no specific trends in the *y*-intercept of the model strategies to match the empirical data.

**Table 1.** Mean (±1 *SEM*) of the coefficients of the linear regression equation best fit to the data of logMAR visual acuity against defocus amplitude for different temporal frequencies of fluctuations. Results from the empirical data and the five computational strategies are shown.

Temporal frequency (Hz)	Fit parameters	Empirical data	Average defocus responder	Least defocus responder	Early defocus responder	Late defocus responder	Mixed responder
0.25	Slope	0.21 ± 0.01	0.39 ± 0.05	0.32 ± 0.02	0.44 ± 0.05	0.44 ± 0.05	0.40 ± 0.02
		(*p* = 0.004)	(*p* = 0.01)	(*p* = 0.01)	(*p* = 0.01)	(*p* = 0.01)	(*p* = 0.003)
	*y*-Intercept	+0.05 ± 0.02	−0.10 ± 0.05	−0.09 ± 0.03	−0.14 ± 0.05	−0.11 ± 0.05	−0.11 ± 0.03
	*R* ^2^	0.99	0.97	0.99	0.98	0.98	0.99

0.50	Slope	0.18 ± 0.17	0.35 ± 0.06	0.24 ± 0.03	0.37 ± 0.06	0.43 ± 0.08	0.34 ± 0.07
		(*p* = 0.01)	(*p* = 0.03)	(*p* = 0.01)	(*p* = 0.03)	(*p* = 0.03)	(*p* = 0.04)
	*y*-Intercept	+0.05 ± 0.02	−0.08 ± 0.07	−0.08 ± 0.03	−0.08 ± 0.07	−0.12 ± 0.09	−0.09 ± 0.08
	*R* ^2^	0.98	0.95	0.97	0.94	0.94	0.91

1.0	Slope	+0.05 ± 0.02	−0.08 ± 0.07	−0.08 ± 0.03	−0.08 ± 0.07	−0.12 ± 0.09	−0.09 ± 0.08
		(*p* = 0.02)	(*p* = 0.09)	(*p* = 0.002)	(*p* = 0.03)	(*p* = 0.04)	(*p* = 0.07)
	*y*-Intercept	+0.06 ± 0.18	−0.06 ± 0.09	−0.07 ± 0.01	−0.05 ± 0.06	−0.01 ± 0.09	−0.05 ± 0.06
	*R* ^2^	0.98	0.95	0.97	0.94	0.94	0.91

2.0	Slope	0.12 ± 0.02	0.19 ± 0.06	0.11 ± 0.02	0.28 ± 0.07	0.28 ± 0.04	0.18 ± 0.01
		(*p* = 0.04)	(*p* = 0.10)	(*p* = 0.04)	(*p* = 0.06)	(*p* = 0.02)	(*p* = 0.003)
	*y*-Intercept	+0.04 ± 0.02	−0.01 ± 0.04	−0.1 ± 0.03	−0.06 ± 0.08	−0.07 ± 0.04	−0.02 ± 0.01
	*R* ^2^	0.93	0.82	0.92	0.89	0.96	0.99

### The 2-Hz, 0.5-D blip in acuity

**Figure 6.**
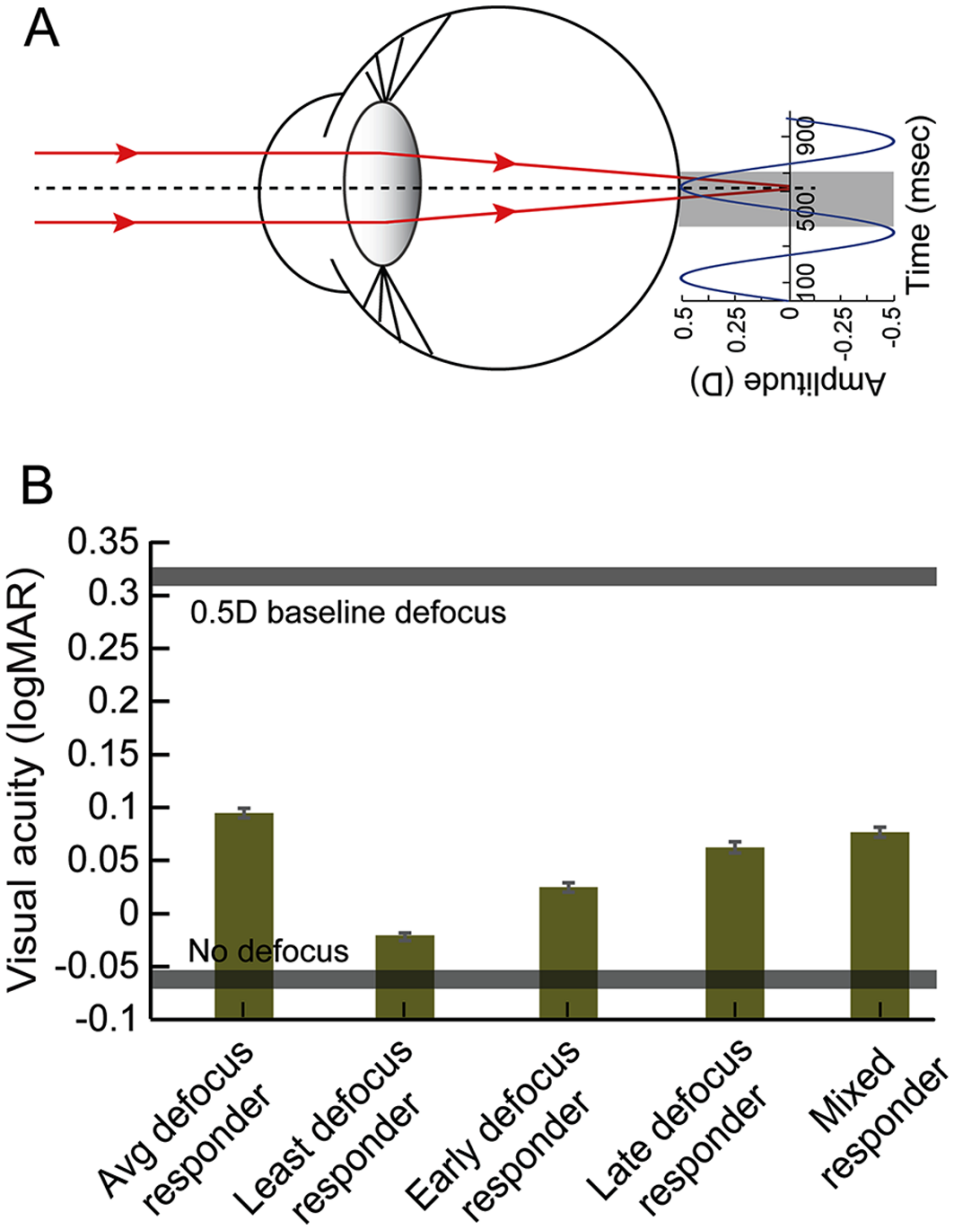
(A) Schematic representation of the impact of 0.5 D of defocus fluctuation at 2 Hz about a baseline defocus of 0.5-D hyperopia. The gray bar in the temporal fluctuations profile is an example of the 300-ms target presentation epoch in each trial of the psychophysical paradigm. (B) Mean (±1 SEM) logMAR visual acuity obtained with a 0.5-D defocus fluctuation at 2 Hz about a 0.5-D baseline value in the five strategies tested in this study. The lower and upper gray bands represent the ±1 *SEM* of logMAR acuity of the model simulations with no defocus and with only 0.5 D of static defocus, respectively.

An intriguing feature in the empirical data was the improvement in logMAR visual acuity and steepening of the psychometric function with 0.5-D defocus fluctuating at 2-Hz temporal frequency relative to the no-defocus condition (Figures 4A and 5A). Such a trend was not observed in any of the computational strategies tested in this study (Figures 4B to 4F and Figures 5B to 5F). To explore putative reasons for this difference in trend, a closer look at the optical conditions that prevailed during empirical testing versus the computational simulations was undertaken. A difference between the two settings was the correction of the residual hyperopic refractive error after cycloplegia in subjects that participated in the empirical study. This hyperopic refractive error (mean ± 1 *SD*, +0.31 ± 0.15 D) remained uncorrected during the empirical testing and this was assumed to be fully corrected during the computational analysis. To determine if this residual hyperopia accounted for the observed improvement in acuity, the template matching analysis was repeated with a residual hyperopic defocus of 0.5 D combined with 0.5 D of temporal fluctuations at 2-Hz frequency (Figure 6A). The 0.5-D residual hyperopic defocus represented the mean + 1 *SD* of the empirical data, and it was chosen to clearly demonstrate its impact on visual acuity with temporal variations in defocus across the different decision-making strategies. All five strategies described earlier were simulated for this combination of defocus, and the results are shown in Figure 6B. Regardless of the strategy adopted, the simulated visual acuity with 0.5-D hyperopia and 0.5-D temporal fluctuations at 2 Hz was always better than the acuity with only 0.5-D hyperopia and no temporal fluctuations (Figure 6B). Among the different strategies, the least defocus responder strategy resulted in acuities closest to the level of no defocus, relative to all other strategies (Figure 6B). Thus, the observed improvement in acuity and steepening of the psychometric function with the 0.5-D–induced defocus at 2 Hz in Figures 4A and 5A could be explained by the canceling out of residual hyperopia in the presence of temporal fluctuations in defocus.

## Discussion

### Summary of the findings

**Figure C1.**
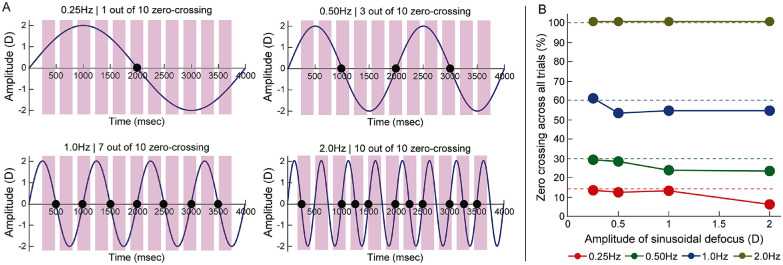
(A) Schematic diagram illustrating the increase in the instances of zero crossings (filled black circles) for 10 discrete epochs of stimulus presentation, each lasting 300 ms, distributed over 4 seconds of sine-wave oscillation. In the actual simulations, the sine-wave oscillation extended continuously over the entire psychophysical procedure containing 110 instances of stimulus presentation. (B) Percentage of zero crossings plotted as a function of the amplitude of sinusoidal defocus for all four frequencies of temporal fluctuations tested in this study. Horizontal lines of the same color indicate the percentage of zero crossings predicted from the probability formula described in the text.

• For the range of defocus amplitudes and frequencies tested and stimulus parameters used (e.g., stimulus presentation duration of 500 ms), the visual acuity for Sloan optotypes deteriorated with increasing defocus amplitude at a rate inversely proportional to the stimulation frequency (Figures 3 and 4; Table 1).
• The empirical trends may be explained by a template matching model of acuity that is governed by the minimum defocus available during optotype display (Figures 1 and 4 to 6; Table 1).
• Acuity loss for a given amplitude and temporal frequency combination of defocus and for 300 ms of optotype display is critically determined by the probability of zero-defocus encounters within the presentation duration (Figures 5 and C1). For the present combination of experimental parameters, the probability of a zero crossing was 100% for the 2-Hz temporal frequency signal (Figure C1). This enabled the minimum defocus decision strategy to minimize acuity loss at this temporal frequency.
• Visual acuity improvement with the 0.5-D, 2-Hz defocus signal in the empirical data relative to baseline may be explained by the temporal defocus minimizing the impact of the ∼0.5-D hyperopic defocus that was left uncorrected in the study following cycloplegia (Figure 6).

### Template matching model of acuity and the decision-making strategies

The present study constructed a template matching model of acuity to explore several decision-making strategies that could be employed by the visual system to optimize vision in the presence of temporal defocus fluctuations (Figure 1). All strategies tested here involved some level of temporal averaging of the blurred retinal image over the 300-ms duration of target presentation (Figures 1 and 2). To average information temporally, this model assumed that the visual system stored information about the target over the entire presentation duration and accessed this information sequence at the time of decision making to implement one of the five decision strategies (Figures 1 and 2). In the least defocus strategy, it was also assumed that the visual system bins the information presented within the presentation epoch into smaller chunks (six 50-ms bins for the 300-ms presentation, in this case) and identified the bin that produced the best image quality for template matching. For operational ease, this strategy was implemented in the present study by choosing the least defocus value among the six bins for template matching (Figure 2). However, given that the visual system may not have direct access to defocus values, the quality of the averaged blurred image from each 50-ms bin will have to be analyzed and the image with the best quality (based on some fixed criterion) will have to be selected for template matching. Alternatively, the averaged blurred image from each 50-ms bin could be subjected to the template matching process, and the decision could be taken from the image that produces the best match from among all averaged images within the presentation epoch. Finally, it was also assumed in four of the five strategies (except the mixed responder strategy) that the decision-making criterion did not vary within or across trials, reflecting consistency of decision-making in human observers throughout the task (Figures 1 and 2). Additionally, in the mixed responder strategy, it was assumed that each of the four decision-making strategies had equal probability of occurrence. Although these assumptions may be too simplistic and should consider added complexities of human decision making (e.g., a priori biases, decision strategies to minimize the loss function) (Gonzalez, Fakhari, & Busemeyer, 2017; Haralick, 1983; Moran, 2015; van Ravenzwaaij, van der Maas, & Wagenmakers, 2012), the present exploration is certainly a starting point to understand decision making that is aimed at optimizing recognition acuity in the presence of temporally varying defocus. During initial exploration, simulations of a least defocus strategy with no temporal averaging were also performed but that strategy was later discarded based on the physiological implausibility of the visual system making a decision based on a single sample point from the entire sequence of stored information (data not shown) (Di Lollo, 1980). A minimum temporal integration time of 33 to 50 ms is needed for detecting simple sine-wave gratings, and this duration might only increase for more complex stimuli such as the Sloan optotypes used in this study (Gorea & Tyler, 1986).

The strategies tested here are not meant to be an exhaustive list of possibilities that the visual system could employ to optimize performance but rather a reflection of some intuitive decision-making strategies that may be hardwired in the human visual system for low-level tasks such as optotype recognition (Green & Swets, 1966; Yu, 2013) (Figure 1). The study qualitatively compared the output of these different strategies with the empirical data trends obtained from human observers (Figures 4 and 5; Table 1). A signature feature of the empirical data that the model simulations sought to replicate was the inverse relation between the loss rate of acuity and the temporal frequency of defocus fluctuation (Figure 4A; Table 1). The early and late defocus averaging strategies could not replicate this trend (compare Figure 4A with Figures 4D and 4E; Table 1), whereas the other strategies did so with different levels of match to the empirical data (Figures 4B, 4C, and 4F; Table 1). Of the three, the potpourri of strategies was expectedly heavily influenced by the trends in the average and least defocus strategies and thus cannot be considered as a unique strategy adopted by the visual system to optimize performance (Figure 4F). These results suggest that the visual system may optimize acuity in the presence of temporally varying defocus by sampling image quality across the entire presentation epoch and choosing the sample region that maximized information about the target presented—in this case, maximizing the spatial frequency content of the optotype by choosing the region of minimum defocus (Figure 1C).

A critical factor that explains the inverse relation between visual acuity loss rate and temporal frequency of defocus is the number of zero-defocus encounters during the 300-ms optotype presentation epoch (Figure C1). The probability of such zero-defocus encounters steadily increases with the temporal frequency of the fluctuation and reaches 100% for frequencies whose time periods between the 0-D instances are shorter than the presentation duration. The time period between 0-D instances is 250 ms for the 2-Hz temporal frequency; therefore, the visual system always encountered a zero-defocus crossing within the 300-ms presentation epoch in this study (Figure C1). The least defocus strategy inherently takes advantage of this trend to minimize the acuity loss across defocus amplitudes by sampling the presentation epoch into short 50-ms bins and choosing the bin that has generated the best image quality within the presentation epoch (Figure 4C). This advantage is progressively lost with a reduction in temporal frequency as the chances of zero-defocus encounters is reduced to only 30% and 15% for the 0.50-Hz and 0.25-Hz signals, respectively (Figure C1). The average defocus strategy is only partially benefited by the increased chances of zero-defocus encounters at higher frequencies, as the decision is based on the average of the entire 300 ms of data containing a range of different defocused images (Figure 4B). The early and late defocus strategies also lose this advantage, for the chances of zero-defocus encounters in the first or last 100 ms of the present epoch are rather slim across all temporal frequencies (Figures 4D and 4E).

The aforementioned analysis leads to two important insights into the present results. First, the visual acuity trends reported here are critically dependent on the relation between the duration of stimulus presentation and the temporal frequencies of defocus fluctuations tested. For any given combination of temporal frequency and amplitude of defocus, the visual acuity loss will become progressively smaller with an increase in target presentation duration due to increased chances of zero-defocus encounters (Figure C1). This loss will asymptote when the target presentation duration exceeds the time period of zero-defocus encounters for that temporal frequency (Figure C1). Unlike experimental settings where target presentation durations are controlled, resolution acuities recorded in the clinic or resolution tasks performed in real life involve processing of visual information that stays on for longer durations (several seconds, typically). Thus, the deleterious impact of temporal fluctuations in defocus for these tasks may be smaller than what is reported in this study (Figures 3 to 5). Second, task performance also worsened with a reduction in temporal frequency of fluctuation, as reflected in the slope of the psychometric functions (Figure 5A). The analysis described above suggests that this loss may not be related to increased difficulty in optotype recognition during individual presentations of the psychometric test but rather due to an increase in the variability of defocus values encountered across presentations within the 300-ms epoch. Lower temporal frequencies will produce larger trial-on-trial variability in defocus than higher temporal frequencies, resulting in greater variability in image quality of the optotypes of a given size for the former than latter frequencies. This variability may translate into progressively shallower slopes of the psychometric function with a decrease in the temporal frequency of defocus fluctuation (Figure 5A).

### Implications for vision loss with accommodative microfluctuations and spasm of near reflex

Unlike the impact of static optical defocus on spatial vision (DeValois & DeValois, 1990; Guirao & Williams, 2003; Legge, Mullen, Woo, & Campbell, 1987; Mon-Williams, Tresilian, Strang, Kochhar, & Wann, 1998), relatively little is known about the impact of temporal variation in optical defocus on visual functions and visual performance. As observed in this study, the extent to which these fluctuations can be ignored or the assumption is valid depends on the amplitude and frequency characteristics of these fluctuations (Figures 3 to 5). Visual acuity remained unaffected with 0.25 D and 0.5 D of defocus at all temporal frequencies tested in the present study (Figure 4), supporting the assumption that defocus fluctuations arising from physiological accommodative microfluctuations (amplitude of 0.25–0.50 D at frequency ≤ 2 Hz) are inconsequential to visual resolution or recognition (Charman & Heron, 2015; Kotulak & Schor, 1986). This is rather expected, as their amplitudes are within the perceptual depth of focus of the visual system (Kotulak & Schor, 1986). However, Winn et al. (1989) demonstrated that these microfluctuations may become perceptually detectable in human observers and that this blur detection may act as an odd-error cue for accommodation, without the need for a subthreshold blur detector (Kotulak & Schor, 1986). Following the observations of Winn et al. (1989), the present study hypothesizes that the perceptual detectability of defocus in the accommodative microfluctuations may arise from their low-frequency components having a larger amplitude than their high-frequency counterparts. Defocus fluctuations in the range observed in the spasm of near reflex (1.5–2.0 D) do have a frequency-dependent negative impact on visual acuity (Figures 3 and 4) (Bharadwaj et al., 2020). This vision loss may largely arise from the low-frequency components of the broadband spectrum for two reasons. First, the amplitudes of the low-frequency components are larger than their higher-frequency counterparts, leading to greater loss of image quality in the former than in the latter (Bharadwaj et al., 2020). Second, the defocus generated by high-frequency components may be minimized through the least defocus decision strategy leading to negligible loss of image quality, all relative to their lower-frequency counterparts (Figure 5). Indeed, the visual acuity loss reported in this study for high-amplitude, low-frequency fluctuations are in the same range as what has been reported for patients with equivalent magnitudes of refractive fluctuations in spasm of near reflex (Ampolu et al., 2022).

### Relative potency of static and temporally varying defocus in deteriorating acuity

**Figure B1.**
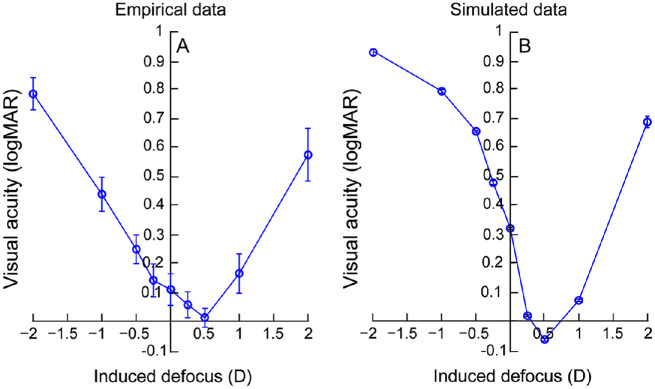
Mean (±1 *SEM*) logMAR visual acuity plotted as a function of induced defocus for empirical data obtained using the ETL (A) and derived computationally using the template matching model of visual acuity described in Figure 1 (B). Positive and negative numbers along the abscissa indicate induced myopic and hyperopic defoci, respectively. For the simulated data, the error bars are smaller than or equal to the size of the symbol in most cases. The overall similarity in the trends of the empirical and simulated data are apparent from this figure.

An interesting prediction arising out of all five defocus averaging strategies tested here is that the visual acuity with temporally-varying defocus will be better than the visual acuity with static defocus of equivalent amplitudes. This is evident when comparing the acuities in the model simulations of static defocus in Figure B1B with those of temporal defocus in Figures 4B to 4F. Data from human observers in Figure B1A (static defocus) and Figure 4A (temporally varying defocus) reflected the same difference, more so for larger defocus amplitudes, confirming the prediction that the former is more potent at reducing acuity than the latter. An implication of this observation is that defocus fluctuations riding over a static defocus may in fact improve acuity relative to when the fluctuations are absent. Indeed, the empirical data for 0.5 D of temporal defocus fluctuating at 2 Hz were statistically significantly better than the baseline condition with ∼0.3 to 0.5 D of hyperopia left uncorrected following cycloplegia (Figure 4A). The model simulations of 2-Hz defocus fluctuations at 0.5-D amplitude supported these trends, more so for the least defocus strategy than others (Figure 6B). In this context, additional questions related to the visual impact of physiological microfluctuations of accommodation arise. For example, do these microfluctuations contribute to an improvement in acuity when they ride over a static defocus? Is the visual acuity with an uncorrected refractive error better with microfluctuations than in their absence? Among other reasons, are the lags or leads of accommodation a result of the microfluctuations optimizing acuity even in the presence of a residual refractive error (Charman & Heron, 2015)? Addressing these questions requires targeted experiments that are beyond the scope of the present study.

## Conclusions

Time-varying defocus deteriorates recognition acuity, with the magnitude of loss being greater for lower than higher temporal frequencies. The visual system optimizes performance by strategically recognizing the target at the point of minimum defocus within the presentation epoch. This strategy mitigates the acuity loss produced by high-frequency fluctuations but not those produced by low-frequency fluctuations. Acuity loss experienced by human observers in the presence of broadband temporal fluctuations (e.g., spasm of near reflex) may thus be explained by the slow, high-amplitude waxing and waning of defocus rather than their higher frequency counterparts.

## Figures and Tables

**Figure A1.**
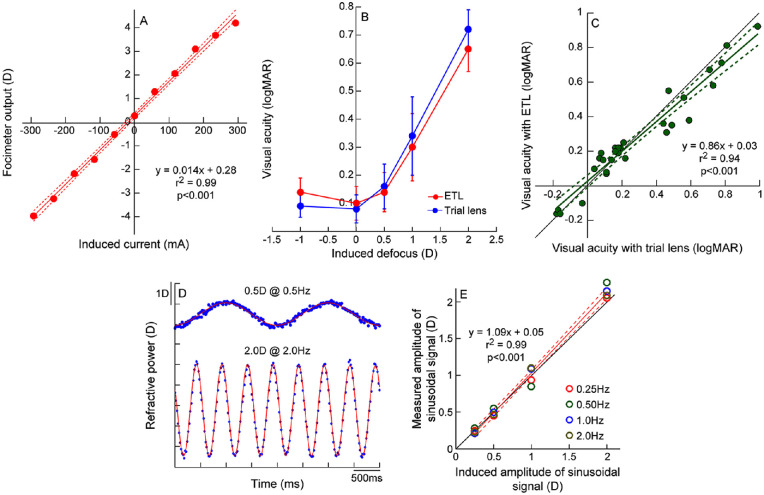
(A) Calibration curve of the ETL used for defoci stimulation in this study. (B) Mean (±1 *SEM*) logMAR visual acuity plotted as a function of induced defocus using standard trial lenses and the ETL. Positive and negative numbers along the abscissa indicate induced myopic and hyperopic defoci, respectively. (C) The same data in panel B plotted as a scatter diagram along with the line of equality (solid black diagonal line). The solid green line and dashed green curves indicate the best-fit linear regression line and the ±95% confidence interval of the fit, respectively. (D, E) Performance of the ETL for sinusoidal oscillations in the electrical current of a given amplitude and frequency. Raw data for refractive power changes were recorded using the photorefractor for 0.5-D oscillations at 0.5 Hz and 2-D oscillations at 2 Hz and are shown in panel B. The solid red curves in this panel show sinusoidal functions that were best fit to these data. Panel E shows the scatter diagram for the measured amplitude of the sinusoidal signal plotted against the induced amplitude of the sinusoidal signal for all four temporal frequencies tested here.

## Appendix A. Appendix A: Calibration and assessment of visual acuity loss with the electrically focus tunable lens

An electrically focus tunable lens (ETL) was used in this study to stimulate varying amplitude and frequency combinations of defoci before the subject. The ETL is comprised of an elastic polymer membrane filled with an optical fluid that changes shape to induce positive and negative power with the electrical activation of a ring seating over this membrane. The lens is controlled through its built-in Lens Driver software (4i) for stimulating varying levels of static or time-varying defoci over a linear operating range of −2 D to +3 D (https://www.optotune.com/el-16-40-tc-lens). The ETL was calibrated over its linear operating range by inputting known values of current (−293 mA to +293 mA, as recommended by the manufacturer, in 58.6-mA steps) and measuring the corresponding dioptric output using a standard clinical focimeter (Figure A1A). The calibration was linear across the entire range of current, with a slope of 0.014 D/mA and *y*-intercept of +0.28 D (Figure A1A). This calibration equation was used to generate all of the sinusoidal defocus profiles used in the main experiment.

Optical quality through the ETL may be somewhat deteriorated from higher-order coma arising from the optical liquid becoming displaced within the elastic membrane, especially in the erect position in which it was used in this study (Suchkov, Fernandez, & Artal, 2019). An additional control experiment was therefore undertaken on six subjects who participated in the main experiment to compare their visual acuity loss with varying magnitudes of defoci (−1 D, 0 D, +0.5 D, +1.0 D, and +2.0 D) induced using the ETL and using standard full-aperture trial lenses from a clinical trial lens case (Figures A1B and A1C). Visual acuity was measured using the same method of constant stimuli psychophysical paradigm as in the main experiment. The overall profiles of acuity loss with both modes of induced defoci were similar, although the acuity loss was slightly smaller with the ETL than with the standard trial lenses (Figure A1B). The latter result is confirmed in Figure A1C, where the slope of the linear regression line of a scatter diagram of acuity losses with the two modes of induced defoci is lower than unity. The 95% confidence interval of this linear regression line, however, overlapped with the 1:1 line, thus the difference in acuity between the two modes of induced defoci was ignored. Overall, the ETL used in this experiment was well calibrated and generated defoci that resulted in acuity loss like that of standard trial lenses used during routine eye examinations.

Finally, the ETL was used in this study for generating temporally varying defocus of a given amplitude and frequency. That the ETL produced defocus values of the desired amplitude and frequency was verified in this study using the following technique. As in the main experiment, the ETL was mounted at 20 mm before the eye of one of the authors of the study, and simulations for 25 seconds generated individual sinusoidal variations for the 16 combinations tested in this study (i.e., 0.25 D, 0.50 D, 1.0 D, and 2.0 D, each at 0.25 Hz, 0.50 Hz, 1.0 Hz, and 2.0 Hz). The participant was cyclopleged using the same technique as the main experiment for this control experiment and fixated on a thin line containing high spatial frequency information at a 2-meter viewing distance. The photorefractor was aligned to the participant's eye and recorded the refractive power change in the eye at 50 fps for each amplitude and frequency combination of defocus for offline analysis. The order of presentation of the amplitude and frequency combinations of defoci was randomized in this control experiment. The raw data of refractive power recorded by the photorefractor were cleaned for blinks and other outliers, smoothed using a 100-ms running-averaging window, and scaled for the defocus calibration factor of this participant (1.12) using MATLAB (Sravani, Nilagiri, & Bharadwaj, 2015). The scaled data were then fitted with a sinusoidal function using the standard nonlinear fitting algorithm in MATLAB to derive the amplitude and frequency of the refractive power fluctuation (Figure A1D). The derived amplitude was then plotted against the input amplitude of fluctuation for each temporal frequency, and a linear regression function with ±95% limits of agreement was fitted to these data (Figure A1E). The slope of this function was 1.09 ± 0.3, with a *y*-intercept of −0.05 and *R*^2^ of 0.99 (Figure A1E). This result indicated that the ETL did produce accurate sinusoidal variations in defocus of the desired amplitude and frequency used in this study.

## Appendix B. Appendix B: Template matching model of acuity for static defocus

The template matching model of acuity described in the main experiment was tested and validated for varying magnitudes of static defoci before it was applied to varying amplitude and frequency combinations of temporal defoci. The steps involved in the construction of the model and generation of the psychometric function were identical to those of the temporally varying defocus signal (Figure 1). All simulations were performed with a baseline hyperopia of 0.5 D, reflecting the magnitude of residual refractive error that was left uncorrected during empirical testing following cycloplegia. However, unlike temporal varying defoci, the defocus signal and thus the degraded retinal image were stable throughout the 300-ms epoch of stimulus presentation. Thus, the different decision-making strategies that were explored for temporally varying defoci in the main experiment do not apply to status defoci, making the latter calculations a lot simpler than the former.

The visual acuity outcomes of this analysis are compared to those of the empirical results obtained in six subjects shown in Figure B1. The overall patterns of acuity loss with varying magnitudes of induced defoci were similar for the empirical data (Figure B1A) and model simulations (Figure B1B) in that (a) the best acuity was obtained with 0.5 D of induced myopia that corrected for the aforementioned residual hyperopic error (Figure B1), and (b) the rates of loss of visual acuity with induced defocus were similar for myopic and hyperopic defoci, about the point of best focus, for both empirical data (myopic defocus: *y* = 0.26*x* – 0.01; hyperopic defocus: *y* = 0.35*x* + 0.08) and simulated data (myopic defocus: *y* = 0.25*x* + 0.03; hyperopic defocus: *y* = 0.30*x* + 0.43) (Figure B1). These results are similar to previously reported losses in visual acuity with induced myopic and hyperopic defoci (Leube, Kostial, Ochakovski, Ohlendorf, & Wahl, 2018). Based on the results, the template matching model was deemed validated for simulating acuity losses with temporal variations in defocus. The absolute magnitude of acuity loss for a given value of induced defocus, however, did not match between empirical and simulated data, as the former resulted in overall better acuity than the latter (Figure B1). Although this difference could be attributed to many sources (e.g., empirical data were measured under polychromatic light and the simulated data were computed for 555-nm monochromatic light) (Ravikumar, Thibos, & Bradley, 2008), neural noise was not manipulated in the simulations to match empirical acuity data as done by Watson and Ahumada (2008); these were ignored as the overall goal of the template matching model was not to generate acuities that matched empirical data.

## Appendix C. Appendix C: Analysis of zero-defocus crossing with sinusoidal variations in temporal defocus

Unlike static defocus, the magnitude of temporal defocus varies with its amplitude and frequency (Figures 1B to 1E). One parameter in the temporal fluctuations that may influence acuity measurements is the probability of the signal having a zero-defocus crossing from positive to negative values or vice versa (Figures 1B to 1E). For example, if the individual uses the least defocus strategy to optimize acuity in the presence of temporal variations in defocus (Figure 1C), the chances of correctly identifying the optotype increase when the stimulus presentation epoch contains a zero crossing relative to when there is no zero crossing. When extended to the complete psychophysical procedure, the proportionality of correct responses for a given optotype size will overall increase with the number of instances of presentations with zero crossings. This may result in better acuity relative to a condition with no or minimal instances of zero crossings.

For a given temporal frequency of a sinusoid (*f*), the time period between instances of a 0-D crossing is 1/2*f*. The probability (*P*) of occurrence of a zero crossing for this temporal frequency within the stimulus presentation epoch (*t* seconds) is therefore given by *P* = *t* × 2*f*, independent of the defocus amplitude. Based on this formula, the probability of a zero crossing is always 100% for temporal frequencies whose time periods between the 0-D instances are shorter than the presentation duration. This probability progressively reduces for temporal frequencies whose time periods between the 0-D instances are longer than the presentation duration. In the present case, the time period between 0-D instances was 250 ms for the 2-Hz temporal frequency, which is shorter than the 300-ms presentation epoch used in this study. Therefore, the probability of a zero crossing within this presentation epoch is always 100% for all defocus amplitudes. Using a similar logic, the probabilities of a zero crossing for 1 Hz, 0.50 Hz, and 0.25 Hz are 60%, 30%, and 15%, respectively (Figure C1). These are represented schematically in Figure C1A by taking 10 discrete 300-ms stimulus presentation epochs over the entire 4-second duration of sinusoidal oscillation (producing one full cycle of the 0.25-Hz temporal frequency signal). Only one stimulus presentation epoch contained the zero crossing for the 0.25-Hz signal, and it steadily increased with temporal frequency to include 10 presentation epochs with zero crossings for the 2-Hz signal (Figure C1A). Stimulus presentation epochs with the 2-Hz signal sometimes contained more than one zero crossing and are not illustrated in this analysis for the sake of simplicity.

These probabilities were verified in the model simulations for all combinations of amplitude and frequency of the temporal defocus signal. Psychometric functions were generated for each amplitude and frequency combination of temporal defocus. For every trial of the psychometric function, a zero crossing within the stimulus presentation epoch was identified if the maximum and the minimum defocus values within the epoch had non-identical polarities; that is, the maximum defocus was positive and the minimum defocus was negative or vice versa, or the maximum defocus had a non-zero value when the minimum defocus was zero, or vice versa. The proportion of zero crossings for a given combination of frequency and amplitude of defocus was then calculated by dividing the number of epochs with zero crossings by the total number of epochs used to generate the psychometric function (Figure C1B). The results largely followed the probability predictions noted above, with minor variations in the probability of zero crossings across different amplitudes occurring by chance. Overall, these results demonstrate why optotype recognition and thus acuity could be better with the 2-Hz signal relative to the lower frequencies for someone employing the minimum defocus strategy for optimizing visual resolution with temporal defocus.
